# Optimizing Wind Power Generation while Minimizing Wildlife Impacts in an Urban Area

**DOI:** 10.1371/journal.pone.0056036

**Published:** 2013-02-08

**Authors:** Gil Bohrer, Kunpeng Zhu, Robert L. Jones, Peter S. Curtis

**Affiliations:** 1 Department of Civil, Environmental and Geodetic Engineering, The Ohio State University, Columbus, Ohio, United States of America; 2 Department of, Evolution, Ecology, and Organismal Biology, The Ohio State University, Columbus, Ohio, United States of America; DOE Pacific Northwest National Laboratory, United States of America

## Abstract

The location of a wind turbine is critical to its power output, which is strongly affected by the local wind field. Turbine operators typically seek locations with the best wind at the lowest level above ground since turbine height affects installation costs. In many urban applications, such as small-scale turbines owned by local communities or organizations, turbine placement is challenging because of limited available space and because the turbine often must be added without removing existing infrastructure, including buildings and trees. The need to minimize turbine hazard to wildlife compounds the challenge. We used an exclusion zone approach for turbine-placement optimization that incorporates spatially detailed maps of wind distribution and wildlife densities with power output predictions for the Ohio State University campus. We processed public GIS records and airborne lidar point-cloud data to develop a 3D map of all campus buildings and trees. High resolution large-eddy simulations and long-term wind climatology were combined to provide land-surface-affected 3D wind fields and the corresponding wind-power generation potential. This power prediction map was then combined with bird survey data. Our assessment predicts that exclusion of areas where bird numbers are highest will have modest effects on the availability of locations for power generation. The exclusion zone approach allows the incorporation of wildlife hazard in wind turbine siting and power output considerations in complex urban environments even when the quantitative interaction between wildlife behavior and turbine activity is unknown.

## Introduction

Many organizations have invested in clean energy or set targets for substituting a percentage of their power generation from renewable sources. Among renewable power sources, wind turbine energy is technologically mature and economically competitive. Typical economic considerations for wind farm locations are driven by long term wind statistics and expected energy output [1]–[4], interactions with other turbines [5], [6], and turbine height [7]. Environmental considerations, including collision risk with birds and bats, also affects the locations for wind farms [2], [3], [8]–[13].

The optimization of wind turbine location in urban areas, where small- and medium-sized wind turbines could be installed by families, organizations, municipalities, or other property owners is particularly challenging. In such limited-space urban applications, possible locations for such turbines are constrained by property boundaries and surrounding structures, particularly buildings and trees that affect wind flow in complex ways both locally and at high resolution [14]–[18]. The challenge often is compounded by difficulty in obtaining high resolution urban maps that include the height and shape of all wind obstructions [19], [20]. Moreover, it is of particular interest to avoid hazards to wildlife, such as bird and bat collisions [8], [21]–[28].

Given these constraints, optimizing turbine location in urban environments requires the incorporation of three disparate types of input data: a map of buildings, roads and other habitat types in the domain of interest, a map of power generation potential, and an assessment of environmental impact for each potential location [11], [26]. In limited-space applications, unlike for large-scale wind farms, the primary goal is not to extract the maximum amount of power from the region, but to find the best location for a limited number of turbines. Therefore, only locations with the best expected power output need be considered, unless other restrictions prevent placing turbines there. To achieve these ends, we propose to combine the application of exclusion zones – locations with the highest wildlife activity, but could include other environmental resources or ecosystem services, with high-resolution wind distribution data. While exclusion and buffer zones based on nature reserves or nesting sites have been proposed and applied before (e.g., [2], [3], [26]), they have never been proposed at high resolution in an urban settings. Using the exclusion-zone approach, the difference between the best power-generation potential across the full siting domain and the highest power generation potential at the remaining, unrestricted areas (after exclusion zones are applied) reflects the cost of avoiding negative environmental impact. As the threshold for acceptable environmental risk decreases, the size of the exclusion zone increases, and the maximal power generation potential in the remaining unrestricted area may either remain unaffected or decrease. The shape and rate of decrease of the maximal power curve with respect to wildlife risk provides a tool to evaluate tradeoffs between wind energy and environmental impact.

Here, we used the campus of the Ohio State University (OSU) in Columbus Ohio, USA, as an example of an urban application. The OSU campus, typical of many urban areas, includes buildings, open spaces, paved surfaces and vegetation. Explicit 3-D surface morphology information, including tree and building locations, height and shape, was obtained by combining publically available GIS data and airborne LIDAR scans of the campus. A high-resolution map of power generation potential throughout the campus was obtained by combining atmospheric large-eddy simulations with the long-term wind climatology. The spatial distribution of birds around campus during the summer season was used as a surrogate for wildlife activity to determine exclusion-zones. We demonstrate how the locations for which wind turbines should be considered to both maximize power generation and minimize collision hazard to wildlife were identified using the exclusion zone approach.

## Methods

### 1. Preparing the 3-D surface morphology

Our goal was to assign a horizontal location, above-ground vertical elevation, and feature code to each 3×3 m cell within the entire OSU campus. This 3×3 m resolution was selected to match the resolution of the atmospheric model. Each cell was characterized as one object type: building, pavement, waterway, tree, or grass.

Data for ground elevation and object locations and shapes came from two sources: (1) Franklin County, Ohio, USA GIS data and (2) Ohio Statewide Imagery Program (OSIP) data. The Franklin county database was obtained from the Franklin County Auditor's office (www.franklincountyauditor.com) and includes data in ArcGIS and AutoCAD DXF formats. OSIP is a data product that was developed from processed airborne LIDAR scans. The OSIP data was downloaded from http://gis3.oit.ohio.gov/geodata/. The OSIP data included the following products: Ground Digital Terrain Model (DTM) source: 5000×5000 ft^2^ (1524×1524 m^2^) tiles of the ground elevations at 1 ft^2^ (0.305×0.305 m^2^) resolution; Original return source: 5000×5000 ft^2^ irregularly dispersed at a distance of approximately 6.25 ft (1.904 m) on average; Background Images: 5000×5000 ft^2^ (1524×1524 m^2^) at 1 ft^2^ (0.305×0.305 m^2^) resolution, color geo-referenced MrSID images. These data sets were referenced using the Ohio State Plane South Nad83 Nav88 Survey feet coordinate system.

**Figure 1:**
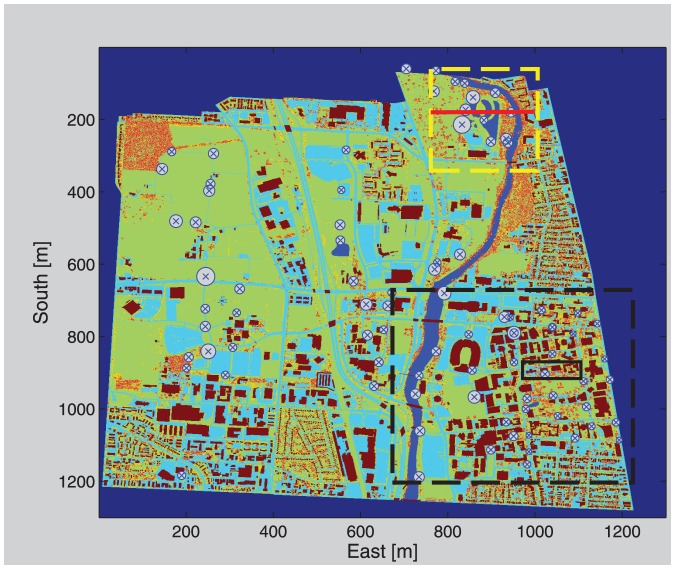
Cell type classification map of the OSU campus, simulation domains, and bird survey locations. Brown cells are buildings, red/orange/yellow are tall, medium and short trees respectively, dark blue is water surface, light blue is paved surface, light green is grass surface. The dark blue region at the outer edges of the map is an unclassified outer-edge buffer. The research wetland and central campus simulation domains are marked with yellow and black dashed frames, respectively. The bold black box marks the section illustrated in Appendix Fig. A.1. The red bar at the wetlands domain marks the location of the vertical cross-section illustrated in Fig. 3. Bird survey locations are marked with an x, and the radius of the circle around these locations is proportional to the total number of species observed at that location. X and Y axis represent eastward and southward coordinates in meters.

We used the GIS data to separate surface objects, such as buildings, roadways, parking lots, sidewalks, and waterways from other background data into a new data set. Polygons including roadway, parking lot, and sidewalk data were assigned properties of “pavement”; building layers formed a single feature type, “buildings”; and rivers, lakes, and ponds formed a single feature type, “waterways”. We extracted tree information from the OSIP LIDAR data by comparing the original return elevations to the processed ground elevations. Cells where the LIDAR returns were at least 3 m higher than the ground elevation and that were not previously classified as buildings were classified as “trees”. All remaining unclassified cells were classified as “grass”. To insure that each GIS cell was only occupied once we created a hierarchy of feature dominance in the order: buildings, trees, pavement, water, and grass. Each cell in the resulting gridded 2D cell-type 3×3 m classification map was defined by its horizontal center and had an associated feature code (Fig. 1). Above-ground elevations were assigned to all features (S. Appendix Fig. A.1). Because trees are porous objects and it is difficult to specify their exact height, we separated tree cells into four bins: 5–10; 10–15; 15–20; and 20–25 meters.

### 2. Producing a Detailed Wind Field

We used an observation-based gridded field of winds from the North American Regional Reanalysis (NARR) [29] from 1979 until 2011. The dataset provides data at 3-hr snapshots at a spatial resolution of 0.3 degrees (roughly 32×32 km^2^), and we used a model grid point (40.0748°N, 83.0896°W) located within the OSU campus.

We used the Regional Atmospheric Modeling System (RAMS)-Based Forest Large Eddy Simulations (RAFLES) [30], [31] to model the wind flow at OSU at high resolution. RAFLES is specifically designed to include the effects of vegetation and trees on wind flow and turbulence inside and above forest canopies. The RAFLES model solves the set of compressible Navier–Stokes equations. The model is forced by a vertical profile of the mean horizontal wind, temperature and humidity, which are also used as the initial condition, and by surface heat and water vapor fluxes. We classified the NARR data for the OSU campus to 3 meteorologically-typical periods, characterized by a distinct combination of atmospheric conditions, (Supporting information, Table S1) and conducted a set of RAFLES simulations for each of these typical periods. Each set was defined by a combination of vegetation density and surface heat flux and included 8 simulations with different wind directions. Simulations were at a resolution of 3×3 or 6×6 m horizontally and 3 m vertically. Buildings and trees were explicitly represented in the simulation domain (Supporting information, Fig. S1). Each model run simulated 2.5 hours for spinup and an additional 30 minutes in which results were analyzed. More details about the simulation and assumptions to reduce the number of simulations needed are provided in the supporting information Appendix S1. Due to the very high computational-time requirements of such a high-resolution model we did not simulate the entire campus environment but focused on two important areas – the central campus and the research wetlands (Fig. 1). Each model simulation provided information about the detailed wind field at one characteristic period and a specific wind direction. We used Monin-Obukhov surface similarity to scale the results of each simulation to different wind speeds within the same characteristic meteorological conditions. We combined all simulation results and scaled them to represent the entire long-term period. This was done by a weighted average of all wind fields. The weight for each windspeed-scale single simulation wind field was calculated based on the observed frequency (from the NARR dataset) of the meteorological conditions and wind direction and speed which that simulation result represented.

### 3. Calculation of wind-power potential at each location

A power curve was fitted into an empirical function, *f*_O_, relating power output to specific mean wind speeds. *f*_O_ is typically provided by turbine manufacturers. Here, we used *f*_O_ of a relatively small, 1kW AWP-3.6 wind turbine (Aerofire Windpower, Lafayette, CO, USA. Power curve provided by www.solacity.com). The averaged potential wind power output of each pixel of the 3-D domain was calculated by integrating the power output, *Wij*, of each wind speed bin:
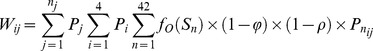
(1)where 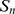 is the horizontal wind speed of the pixel, 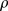 is the air density factor, 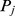 is the probability of meteorological forcing in the historical dataset falling into each simulation category (Supporting information, Table S2), 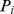 is the probability of wind in that category blowing to one of the four direction bins, 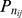
is the probability of the wind in that category and direction to be within a specific range (bin) of speed, and 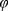 is the turbulence factor. We used our simulation results to scale 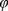 at different locations. The mean relative turbulence level aloft (above 30 m) was assumed to scale as a turbulence factor of 5% and the relative turbulence level at the most turbulent places, such as in the wake of trees, scaled as a turbulence factor of 20%. All other values scaled linearly, between these two levels, based on the relative turbulence at each location.

### 4. Bird surveys

Bird observations were made at survey points every 200 m along transects running across OSU property (Fig. 1). We did not require a permit because no vertebrates were captured, handled or disturbed in this study. Birds were passively observed from public, urban areas where pedestrian traffic other than birdwatchers is common, and were not disturbed in any direct or indirect way by the observers. Observations were made for five minutes at each survey point by experienced avian biologists who recorded the species of bird seen or heard and the number of individuals of each species. We used all observations within 30 m (estimated using streets and buildings as visual references). This distance is approximately the size of a typical patch in the dense urban setting of this experiment. Each survey point was visited at least four times between June 14^th^ and September 3^rd^, 2010, a period that included the peak activity season for local and summering birds in an effort to capture the maximal estimate for bird densities. Observations made during both the early and late morning. To characterize the survey environment, a 30 m-radius buffer circle was made around each survey point on the patch-type map. Then, the occurrence of each land-use patch type falling into the buffer circle was counted, yielding a relative density of each patch type for each bird-survey point.

To extend the point observations of bird species richness and numbers of individuals to the entire campus area, we related the locations of bird observations to the specific environmental patch types around the survey point and fit an empirical model of bird numbers or species richness as a function of the environmental patch type. A stepwise forward multi-variant regression was used to find which of the patch types was significantly associated with larger numbers of individuals or species of birds (Table 1). Models were fitted for each of the four bird survey variables: (1) the mean number of total individuals; (2) mean number of native individuals; (3) mean number of total species; (4) mean number of native species. Native species often are the focus of conservation efforts while non-native species may be present in large numbers but generally are not considered of special conservation concern.

**Table 1:** Empirical equations and line-fit statistics relating the observed number of individual birds or species with the density of the different patch types surrounding each location of the grid.

Bird Variables	Line-fit coefficient by Patch Type					Line-fit Const.	*R^2^*	*P* value
	Grass	Water	Pavement	Building	Tree			
All Species	0.020	1.700				3.650	0.24	<0.001
Native Species	0.017	1.899				2.789	0.19	0.001
(All Individuals)^0.5^			−0.016		−0.022	5.223	0.08	0.026
ln(Native Individuals)			−0.008	−0.030		2.537	0.18	0.011

Only significant model results are shown.

### 5. Exclusion zones and unrestricted-domain power calculation

The exclusion zone is an area that must be protected and therefore is excluded from consideration as a turbine site. Exclusion zones are determined as a prescribed fraction of the total domain with the highest wildlife activity density, or individual numbers or species richness endangered-species abundance. In this study we used the summertime activity of birds as an example for generating exclusion zone considerations. The mean power generation potential at the top (strongest power) 10% of the remaining un-excluded area provided a quantitative metric of the effect of the exclusion area, and we considered it as the power potential of the domain with a given exclusion level. We use the top 10% cutoff rather than the best point in the un-excluded domain because of the need for a large area from which placement could be selected. This is because other restrictions than bird presence or wind could prohibit construction of a turbine at a given site. We tested the effects of different exclusion thresholds of acceptable environmental risk by incrementally increasing the exclusion fraction.

## Results and Discussion

### High-resolution wind distribution and its impact by buildings and vegetation

**Figure 2:**
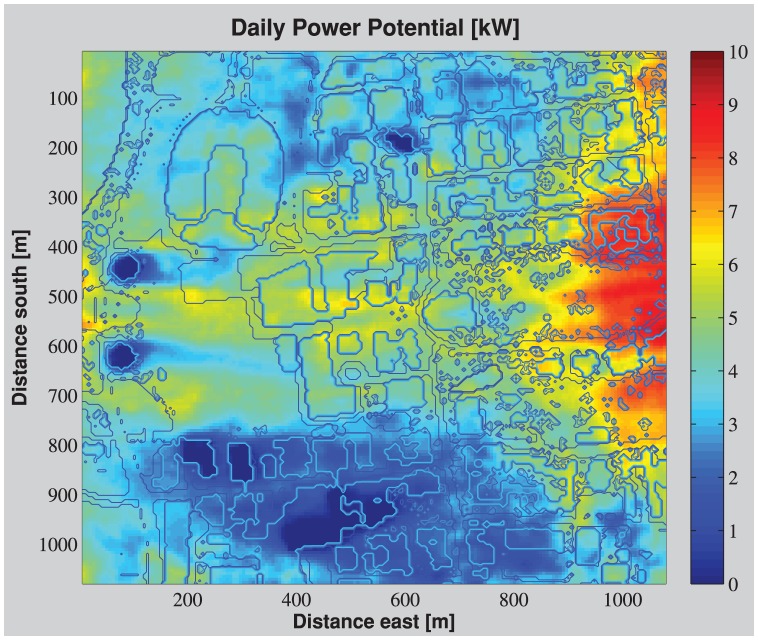
Daily power generation potential of the central campus area (black dashed frame in Fig. 1). Colormap shows the expected mean daily power potential [kW], dark blue lines mark the edges of land-use types and light blue lines mark the edges of buildings. X and Y axis represent eastward and southward coordinates in meters.

**Figure 3:**
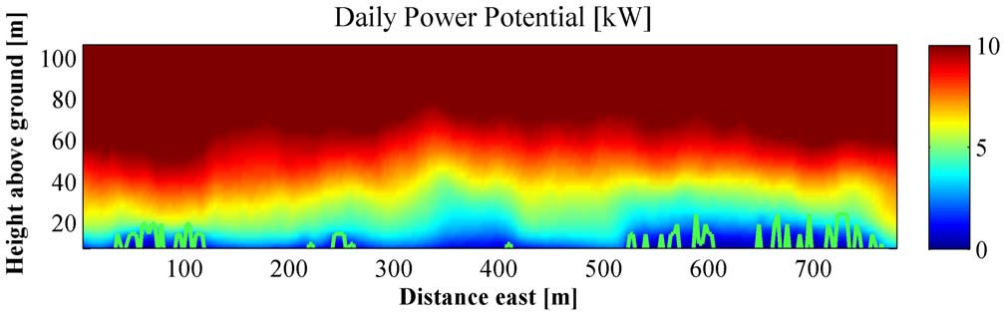
Vertical distribution of daily wind power generation potential over the research wetland (yellow dashed frame in Fig. 1). This plot illustrates power generation at different heights over the cross-section illustrated as a red line in Fig. 1. Green lines mark the upper outline of trees and building. Stronger power potential can be found at lower elevations over open areas. However, as can be noticed by the difference between power over the trees at 100 m east and the shorter trees over 300 m east, the complex 3-D structure affects the height to which the reduction of power potential extends vertically over an obstacle. X and Y axis represent eastward and southward coordinates in meters.

The 3-D map of power generation potential on selected areas on the OSU campus is shown in Figs. 2 & 3. Trees and buildings form a “wind shadow” effect, with weaker wind at lower elevations, near the canopy, relative to the same elevation over open areas (e.g., grass, water, and paved surfaces). However, due to the complex 3-D structure of the obstacles and their interactions, these wind shadow patterns do not follow an easy-to-define distribution downwind of each obstacle. Buildings tend to have a more pronounced downwind shadow than do trees as they block and deflect the wind more efficiently. This is even more pronounced in the neutral cases where surface heat fluxes do not play a role (data not shown).

It is interesting to note that in the Central Campus area (Fig. 2), the grass covered park-like area centered at 900 m east and 550 m south from the northwest corner of the domain in Fig. 2 has the highest wind power potential. The tall buildings of the medical center at the southwest region of the central campus block the wind and lead to a low wind power potential area. An interesting effect is generated by the two tall student dormitory towers at 450 and 650 m south and 100 m east (Fig. 2). The towers slow wind speed immediately above them, but funnel easterly and westerly winds to the grassy gap between the towers, and create a narrow corridor of relatively strong wind. The vertical profiles of power potential (Fig. 3) show that within 10 to 30 m above the ground, the power potential is almost double over open areas than over buildings or trees.

### 1 Bird distribution

The relationship between bird survey variables and map patch types is shown in Table 1. On average we observed 12 species, 8 of which native and 28 individuals, 10 of which native at each observation location/time. The most abundant species included Canada goose and house sparrow. The presence of water had a strong positive effect on bird diversity and increased the predicted numbers of native and total bird species, but did not have a significant effect on the total number of individuals. Paved areas had a negative effect on individual numbers but not on species richness. Buildings had a negative effect on native species individual numbers, but not on total bird numbers. Surprisingly, trees had a negative effect on total numbers, but not on native species numbers. This is probably due to the large numbers of “city” birds, such as doves and sparrows that tend to be common in large numbers around buildings, and few other species, such as Canada geese that aggregate in large numbers on open grass lots.

### 2. Minimizing bird collision risk – the exclusion zone approach

The goal of this study was to demonstrate how high resolution maps, wildlife activity density estimates and wind simulations could be combined to provide placement guidance for wind turbines in urban settings. We did not attempt to produce an operational risk-assessment map for the OSU campus. Extended survey that will include the night time bird and bat activity, and surveys during migration seasons will be needed in order to produce an accurate environmental assessment of wildlife activity and potential risk (see [32], [33] for the importance of OSU campus habitats during migration stopover).

We combined high-resolution data of different types for our urban study area using the exclusion zone approach. At the large (national, state) scale exclusion zones were proposed that restrict wind-power development in and near protected areas [2], [3], [11]. However, limited-domain, small-scale application represent a very different case. Distinct nature-conservation areas or major nesting colonies are typically not included anywhere in the domain, while a buffer distance of a few km from any bird habitat location will incorporate the entire domain. The best location for a wind turbine, while considering wildlife risk in a limited space of an urban or rural community, will have high wind power potential and low risk of bird mortality. Unfortunately, wildlife mortality rates due to wind turbines are known to be location and species specific [8], [22], [25]–[28]. Additionally, it is impossible to predict actual mortality rates before well-parameterized models of bird (or bat) movement that includes their location, height and activity [23], [34]–[36] exist for all species in the area. To avoid this complication, we assumed that risk would be proportional to bird activity density at a given location and used the bird activity during the summer season as an example.

The risk-density – proportionality assumption is commonly used in generating risk-assessment maps [37], [38]. The assumption that density of bird activity at the area around the zone of the potential wind-turbine blades is proportional to the collision risk is implicit in almost any flight model study that attempts to relate flight behavior to collision risk without actual mortality data [23], [34], [35]. Studies that used collision-mortality observations to evaluate this assumption provide contradictory evidence – some found support to the positive relationship between abundance and/or activity density and mortality [8], [39], [40], while others find poor relationships between bird density and mortality [41]. Some studies reported species specific effects, with a significant relationship between abundance and mortality in some species but not others at the same locations [42], [43]. This suggests that the species flight behavior and possible avoidance capabilities play an important role [40], [44], [45]. However, generating exact species-specific predictions for all species would not be feasible, particularly in a complex urban domain. We therefore accept the simple proportionally assumption as a practical solution.

**Figure 4:**
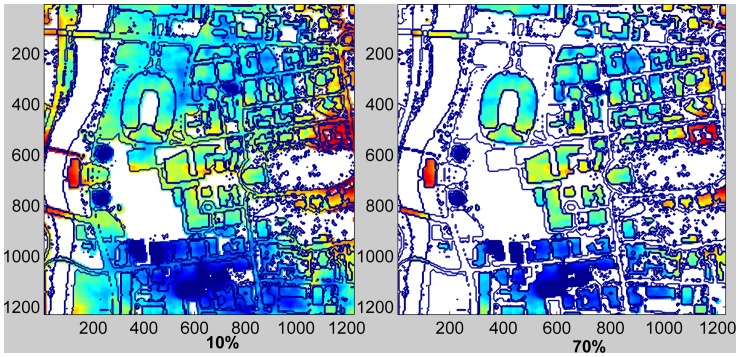
Effects of exclusion zones on expected power generation. The year-round power map at 30 m above ground (red-high, blue–low) overlaid on the map outline of the central campus area. Exclusion areas, 10%, and 70% of total domain with highest native bird density, are marked white.

We then considered the effect of varying the size of conservation exclusion zones on power output. At the “conservative” extreme case, we excluded all areas where bird numbers or species richness was higher than the 10^th^ percentile, i.e. turbine placement was considered only in areas where bird numbers/richness were at the lowest 10%. We then relaxed this requirement in consecutive steps of 10% ending with a case that excluded only 10% of the area where bird numbers/richness was highest (Fig. 4).

**Figure 5:**
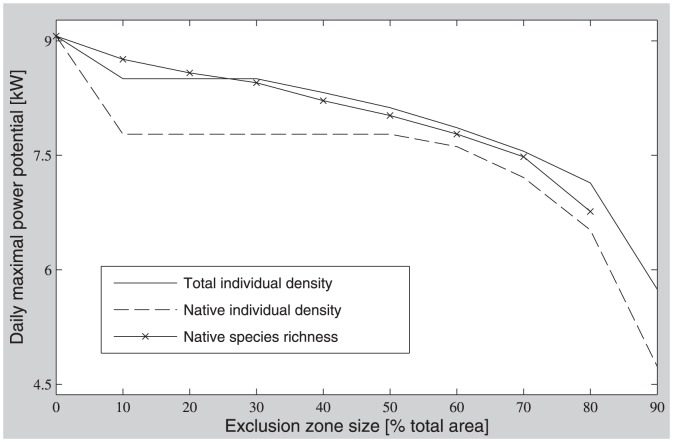
The effect of the relative area of exclusion zones on the maximal daily-mean power generation potential in the best remaining (un-excluded) suitable sites. Suitable sites are calculated as the 10% of the domain area with highest power potential.

As exclusion zones widen, the power generation potential of the top 10% of available locations decreases because more and more of the locations with high power generation potential are excluded. This decrease is non-linear when exclusion is based on number of individuals (Fig. 5). A sharp decrease (8% and 15% in total and native individual numbers, respectively) is caused by exclusion of the first 10% of the domain with the highest bird numbers or species richness. This is because the large grassy parks and the open water areas are the first to be excluded and also tend to have the highest power generation potential because of low drag and lack of obstructions. However, additional enlargement of the exclusion zone up to about 30–50% does not result in a large decrease of power generation potential at the best remaining non-excluded locations. These are typically found above low buildings, parking lots, and roads. This is not the case for species richness-based exclusion, however. In this case, a near linear decrease of power in the best remaining sites is driven by an increase in exclusion zone area.

Our bird observations were limited in space and time as is typical for direct observation animal surveys. Further developments in GPS-based tracking technology [46], particularly in miniaturization that will allow the tracking of small bird and bats is needed in order to incorporate the movement patterns and altitude of wildlife activity in future risk assessments and exclusion-zone considerations. Reduction of the tag prices will make it feasible to track many individuals in a single study and may facilitate wildlife tracking as a risk-assessment tool. Track annotation of birds and bats with turbulence and weather conditions [47]–[50] will allow turbine location decisions to incorporate the full tracks of birds and the behavioral rules according to which birds choose flight tracks and roost locations. These developments could yield more accurate movement models that could be applied at high enough resolution to be relevant to urban spaces.

### Conclusions

We provide an example of a comprehensive data resource to support wind turbine placement decisions in a limited-space urban domain. Such decision support need is typical for university campuses, industrial complexes, farm cooperatives, or other entities that are considering adding wind turbines but want to minimize modification of the landscape in optimizing power generation. We used a large-eddy simulation (LES) in the context of fine resolution wind simulation with both vegetation canopy and buildings. The simulation result has many appealing features for future research. LES quantifies the explicit spatial effects of buildings and vegetation within the domain of a wind turbine. We showed that complex interactions between obstacles in different wind directions lead to a non-linear and complex patterns of wind speed at different heights above ground. LES results provide the information needed to find the location, as well as optimize the height, of the wind turbine. Alternatively, simpler foot-print models can provide information about the wind-shade of each obstruction given the wind speed and direction. While this will neglect the complex interactions between multiple obstructions, it will relax the need for a computationally expensive simulation and will allow resolving many more cases of different weather forcing.

Our study indicates a practical way of balancing the small-scale production of wind energy and minimizing wildlife collision risk. Combining the 3-D potential power generation map with the environmental-impact map leads to a location priority map that will provide planners with information on both the power output and environmental risk of a turbine application, with which they would optimize turbine location and height. In our example, areas supporting above-median bird numbers overlap with the areas where bird species richness is in the top 10–20 percentiles. Our analysis predicts that these areas could be excluded with only small consequences to the expected power generation potential at the best remaining locations.

## Supporting Information

Appendix S1 **Details of the numerical large-eddy simulations used to determine the wind field over sub-domains of the campus and its interactions with buildings and vegetation.** The details of the approach we used to calculate a detailed, high-resolution 3D vegetation cover and building map. The long term wind climatology over the campus and assumptions takes to simplify the full range of meteorological conditions to a small sub-set of representative simulations.
(DOCX)

Table S1
**Wind Statistic over the OSU campus – the probability of meteorological forcing in the historical dataset falling into the simulation category,** 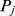 **.** The breakup to three simulation categories was used in the wetland sub-domain simulations. In the central campus, no distinction was made between summer and winter and forcing conditions were categorized as either convective or neutral.(DOCX)

Table S2 **Canopy parameters used in the 3 types of RAFLES simulations.**
(DOCX)

Figure S1 **Illustration of the building and tree processing methods.** (a) Raw lidar point cloud, colored according to patch type (trees in dark green, grass in green and building in gray). (b) Tree and building heights are reassigned, tree pixel are sub-classified according to height.
(EPS)
