# Supplementary material for: Optimizing Wind Power Generation while Minimizing Wildlife Impacts in an Urban Area
Source: PLoS One. 2013 Feb 8;8(2):e56036. doi: 10.1371/journal.pone.0056036 (PMC3568073; doi:10.1371/journal.pone.0056036)
Supplement: Appendix S1 — Details of the numerical large-eddy simulations used to determine the wind field over sub-domains of the campus and its interactions with buildings and vegetation. The details of the approach we used to calculate a detailed, high-resolution 3D vegetation cover and building map. The long term wind climatology over the campus and assumptions takes to simplify the full range of meteorological conditions to a small sub-set of representative simulations. (DOCX) [file pone.0056036.s001.docx]

**Supporting information Appendix S1**

Calculating a Detailed, High-Resolution Wind Field Using Large Eddy Simulations

The Regional Atmospheric Modeling System (RAMS)-Based Forest Large Eddy Simulations (RAFLES) ([Bohrer et al. 2008](#_ENREF_2), [Bohrer et al. 2009](#_ENREF_1)) was used to model the wind flow at OSU at high resolution. RAFLES solves the set of compressible Navier–Stokes equations. The model is forced by a vertical profile of the mean horizontal wind, which also is used as the initial condition. Wind forcing is prescribed by Newtonian nudging. Additional initial conditions are provided in the form of vertical profiles of temperature, pressure, and humidity. It uses periodic boundary conditions at the lateral boundaries and a reflective top with Rayleigh friction at the top boundary. Free slip is assumed at the ground surface and drag force is added to resolved and sub-grid-scale velocities at the bottom boundary and within tree and building domains.

RAFLES' unique numerical mesh formulation includes the effects of hard surfaces in the simulation domain, such as tree stems and buildings, using the shaved grid-cell method. In this method the effective volume and aperture of each grid cell that includes a hard vertical surface is reduced to represent the blockage of the free space to air flow. In tree cells, we used empirical relationships between tree height and stem volume to determine the blockage of volume and aperture. We assume buildings represent a 95% reduction of the open aperture and volume. This was adopted instead of a 100% to prevent numerical instability at the lower corners of a tall, fully solid wall. This is equivalent to assuming that buildings include a few open windows.

### Vegetation and Surface Energy Forcing

The 3-D simulation domain was built from the obstruction map (Fig. 1) with a modified version of Virtual Canopy Generator (V-CaGe) ([Bohrer et al. 2007](#_ENREF_3)). Instead of randomly generating locations of trees in a canopy (as in the standard version of V-CaGe), the modified generator read the cell type classification map, and assigned the specific canopy patch type and height to the generated canopy map. The data from the airborne LIDAR point-cloud was then processed into 3-D structures and vegetation shapes (for example, Fig. S1). Additional vegetation parameters required by V-CaGe, i.e. leaf area index (LAI) of trees of different heights, vertical profile of leaf density, and tree-stem volume, are based on observations made at the research wetland during spring 2010 and winter 2011 using a ground-based portable canopy LIDAR ([Parker et al. 2004](#_ENREF_5), [Hardiman et al. 2011](#_ENREF_4)). Surface forcing for the atmospheric model, i.e. surface emissions of latent and sensible heat fluxes and vegetation albedo, was based on eddy-covariance measurements during 9/2010-6/2011 at the wetland.

**Figure S1.** Illustration of the building and tree processing methods. (a) Raw lidar point cloud, colored according to patch type (trees in dark green, grass in green and building in gray). (b) Tree and building heights are reassigned, tree pixel are sub-classified according to height.

A 3-D domain of 1300 × 1300 pixels, at 3x3x3 m^3^ resolution was generated by V-CaGe and represented the entire OSU campus and surrounding urban area. Due to the simulation capability and efficiency at the Ohio Supercomputer Center, only two, smaller sub-domains were selected. Large eddy simulations were conducted only for the sub-domains. The first one included 300×300 pixels (900×900 m^2^) around the research wetland where land cover was characterized by a mixture of water, trees and grass with a few buildings. The second simulation domain included the OSU central Campus where land cover is predominantly buildings and pavement, with some forest patches and grass-covered green areas and a section of the Olentangy River. This simulation domain had a reduced resolution (6×6×3 m^3^) in order to be able to include an area (1800×1800 m^2^) large enough to encompass the entire central campus.

Simulations were run for different wind directions, meteorological conditions and seasons (see below). Each simulation included 3 or 6 hours (depending on the meteorological conditions) and only the last 30 min of the simulations were analyzed. Across this simulation time span, the effect of the horizontally homogeneous initial condition became negligible and the simulation approached a stationary state with regard to turbulence statistics.

### Assumptions and Simplifications Implemented in Meteorological Forcing Conditions

The long-term wind data from the NARR (http://www.emc.ncep.noaa.gov/mmb/rreanl/) grid-point closest to the campus was divided into the same categories of summer-convective, summer-neutral, winter-convective and winter-neutral. The summer is defined as the time period when trees have leaves: May - October. The winter-convective case was very rare and was ignored. The distribution of wind speed within each category, at each of the cardinal directions was calculated. This was done using histograms with 42 wind speed bins of 0.25 m/s.

Three sets of canopy and meteorological-forcing parameters were used to represent different weather/season conditions. Each season/weather condition was represented by a different leaf area index, reflection coefficient (Albedo), surface sensible heat flux and surface latent (water vapor) heat fluxes. See Table S2 for parameter values used in RAFLES to represent each condition.

Considering all possible combinations of wind speed, direction, surface heat flux and seasons would have demanded a near infinite number of simulation cases. Some assumptions and simplifications were made to reduce the number of simulations to a feasible set that included three representative season/weather-condition cases.

One assumption was that the simulation results for wind speed can be expressed in normalized, unitless terms, as the ratio between the actual mean wind speed at each point at the domain, and the mean wind speed over the domain at a reference height of 35 m above the ground. This assumption is common to analysis of LES results. In this way we limited wind forcing to a single representative vertical profile but were able to scale the normalized results to represent many above-ground wind conditions within the operational range of the turbine.

The next simplification was that wind direction can be represented by only four cardinal directions of north, south, east, and west. When the mean above ground wind direction was other than one of these four, it was approximated to the closest cardinal direction. This allows only four simulations to represent all possible wind directions.

Additional simplifications reduce the state of the atmospheric boundary layer and surface heat flux to three categories: convective, near-neutral, and stable. RAFLES is not well suited to resolve stable boundary layer conditions. However, stable conditions are typically not supportive of power generations, and were therefore ignored. Forcing is thus reduced to only two boundary layer classes: convective and neutral. We further assumed that convective cases are not important during the winter due to snow cover on the ground (which leads to neutral or stable conditions). Finally, we assumed that seasonal differences in leaf density and Bowen ratio (the ratio between sensible and latent heat flux) are represented by the two seasonal peaks of summer and winter. Based on these, an initial driving wind of 1.6 m/s is applied to each simulation, and four simulations with different wind directions are applied to each boundary-layer/season case.

A neutral boundary layer happens when the heat flux from the surface is close to zero. Therefore, differences in wind flow between winter and summer convective simulations are mostly due to differences in leaf drag and are not affected by differences in surface flux forcing. Therefore, we can further reduce the simulation numbers over the central campus sub domain because this area is mostly covered by buildings and pavement and does not have many tree areas. We therefore assume that in the building-dominated Central Campus area the neutral case represents both summer and winter. Table S1 shown the number of 3-hourly NARR data points that fit within each case.

**Table S1.** Wind Statistic over the OSU campus – the probability of meteorological forcing in the historical dataset falling into the simulation category, $P_{j}.$ The breakup to three simulation categories was used in the wetland sub-domain simulations. In the central campus, no distinction was made between summer and winter and forcing conditions were categorized as either convective or neutral.

| Category | Records | North | East | South | West |
| --- | --- | --- | --- | --- | --- |
| Summer convective | 16190 (17.31%) | 30.09% | 33.94% | 19.83% | 16.15% |
| Summer neutral | 30914 (33.06%) | 31.93% | 32.21% | 19.67% | 16.20% |
| Winter | 46400 (49.62%) | 30.92% | 36.88% | 18.50% | 13.70% |
| Total | 93504 |  |  |  |  |

**Table S2.** Canopy parameters used in the 3 types of RAFLES simulations.

| Patch type | Parameter | Summer convective | Summer neutral | Winter |
| --- | --- | --- | --- | --- |
| Water | Mean LAI | 0.01 | 0.01 | 0.01 |
|  | Mean Albedo | 0.8 | 0.8 | 0.8 |
|  | Mean Total Flux | 140 | 10 | 10 |
|  | Mean Bowen Ratio | 0.34 | 0.34 | 0.34 |
| Pavement | Mean LAI | 0.01 | 0.01 | 0.01 |
|  | Mean Albedo | 0.7 | 0.7 | 0.7 |
|  | Mean Total Flux | 140 | 10 | 10 |
|  | Mean Bowen Ratio | 25 | 25 | 25 |
| Grass | Mean LAI | 1 | 1 | 0.01 |
|  | Mean Albedo | 1.34 | 1.34 | 1.34 |
|  | Mean Total Flux | 140 | 10 | 10 |
|  | Mean Bowen Ratio | 0.87 | 0.87 | 0.87 |
| Short trees | Mean LAI | 3 | 3 | 0.01 |
|  | Mean Albedo | 0.14 | 0.14 | 0.14 |
|  | Mean Total Flux | 140 | 10 | 10 |
|  | Mean Bowen Ratio | 0.73 | 0.73 | 0.73 |
| Tall trees | Mean LAI | 4 | 4 | 0.01 |
|  | Mean Albedo | 0.14 | 0.14 | 0.14 |
|  | Mean Total Flux | 140 | 10 | 10 |
|  | Mean Bowen Ratio | 0.73 | 0.73 | 0.73 |
| Buildings | Mean LAI | 0.01 | 0.01 | 0.01 |
|  | Mean Albedo | 0.7 | 0.7 | 0.7 |
|  | Mean Total Flux | 140 | 10 | 10 |
|  | Mean Bowen Ratio | 25 | 25 | 25 |

References

Bohrer, G., G. G. Katul, R. L. Walko, and R. Avissar. 2009. Exploring the effects of microscale structural heterogeneity of forest canopies using large-eddy simulations. Boundary-Layer Meteorology **132**:351–382.

Bohrer, G., R. Nathan, G. G. Katul, R. L. Walko, and R. Avissar. 2008. Effects of canopy heterogeneity, seed abscission, and inertia on wind-driven dispersal kernels of tree seeds. Journal of Ecology **96**:569–580.

Bohrer, G., M. Wolosin, R. Brady, and R. Avissar. 2007. A Virtual Canopy Generator (V-CaGe) for modeling complex heterogeneous forest canopies at high resolution. Tellus Series B-Chemical and Physical Meteorology **59B**:566-576.

Hardiman, B. S., G. Bohrer, C. M. Gough, C. S. Vogel, and P. S. Curtis. 2011. The role of canopy structural complexity in wood net primary production of a maturing northern deciduous forest. Ecology **92**:1818–1827.

Parker, G. G., D. J. Harding, and M. L. Berger. 2004. A portable LIDAR system for rapid determination of forest canopy structure. Journal of Applied Ecology **41**:755–767.
