# Supplementary material for: Optimizing Wind Power Generation while Minimizing Wildlife Impacts in an Urban Area
Source: PLoS One. 2013 Feb 8;8(2):e56036. doi: 10.1371/journal.pone.0056036 (PMC3568073; doi:10.1371/journal.pone.0056036)
Supplement: Table S1 — Wind Statistic over the OSU campus – the probability of meteorological forcing in the historical dataset falling into the simulation category, . The breakup to three simulation categories was used in the wetland sub-domain simulations. In the central campus, no distinction was made between summer and winter and forcing conditions were categorized as either convective or neutral. (DOCX) [file pone.0056036.s002.docx]

| Category | Records | North | East | South | West |
| --- | --- | --- | --- | --- | --- |
| Summer convective | 16190 (17.31%) | 30.09% | 33.94% | 19.83% | 16.15% |
| Summer neutral | 30914 (33.06%) | 31.93% | 32.21% | 19.67% | 16.20% |
| Winter | 46400 (49.62%) | 30.92% | 36.88% | 18.50% | 13.70% |
| Total | 93504 |  |  |  |  |
