# Supplementary material for: Optimizing Wind Power Generation while Minimizing Wildlife Impacts in an Urban Area
Source: PLoS One. 2013 Feb 8;8(2):e56036. doi: 10.1371/journal.pone.0056036 (PMC3568073; doi:10.1371/journal.pone.0056036)
Supplement: Table S2 — Canopy parameters used in the 3 types of RAFLES simulations. (DOCX) [file pone.0056036.s003.docx]

| Patch type | Parameter | Summer convective | Summer neutral | Winter |
| --- | --- | --- | --- | --- |
| Water | Mean LAI | 0.01 | 0.01 | 0.01 |
|  | Mean Albedo | 0.8 | 0.8 | 0.8 |
|  | Mean Total Flux | 140 | 10 | 10 |
|  | Mean Bowen Ratio | 0.34 | 0.34 | 0.34 |
| Pavement | Mean LAI | 0.01 | 0.01 | 0.01 |
|  | Mean Albedo | 0.7 | 0.7 | 0.7 |
|  | Mean Total Flux | 140 | 10 | 10 |
|  | Mean Bowen Ratio | 25 | 25 | 25 |
| Grass | Mean LAI | 1 | 1 | 0.01 |
|  | Mean Albedo | 1.34 | 1.34 | 1.34 |
|  | Mean Total Flux | 140 | 10 | 10 |
|  | Mean Bowen Ratio | 0.87 | 0.87 | 0.87 |
| Short trees | Mean LAI | 3 | 3 | 0.01 |
|  | Mean Albedo | 0.14 | 0.14 | 0.14 |
|  | Mean Total Flux | 140 | 10 | 10 |
|  | Mean Bowen Ratio | 0.73 | 0.73 | 0.73 |
| Tall trees | Mean LAI | 4 | 4 | 0.01 |
|  | Mean Albedo | 0.14 | 0.14 | 0.14 |
|  | Mean Total Flux | 140 | 10 | 10 |
|  | Mean Bowen Ratio | 0.73 | 0.73 | 0.73 |
| Buildings | Mean LAI | 0.01 | 0.01 | 0.01 |
|  | Mean Albedo | 0.7 | 0.7 | 0.7 |
|  | Mean Total Flux | 140 | 10 | 10 |
|  | Mean Bowen Ratio | 25 | 25 | 25 |
